# Commentary: Flow-controlled ventilation maintains gas exchange and lung aeration in a pediatric model of healthy and injured lungs: a randomized cross-over experimental study

**DOI:** 10.3389/fped.2023.1122434

**Published:** 2023-06-02

**Authors:** Dietmar Enk, Patrick Spraider, Julia Abram, Tom Barnes

**Affiliations:** ^1^ Faculty of Medicine, University of Münster, Münster, Germany; ^2^ Department of Anesthesia and Intensive Care Medicine, Medical University of Innsbruck, Innsbruck, Austria; ^3^ Faculty of Engineering and Science, University of Greenwich, London, United Kingdom

**Keywords:** flow-controlled ventilation, respiratory mechanics, gas exchange, pediatric model, dead space effect

**A Commentary on:**
Flow-controlled ventilation maintains gas exchange and lung aeration in a pediatric model of healthy and injured lungs: a randomized cross-over experimental study By Enk D, Spraider P, Abram J, Barnes T. (2023). Front. Pediatr. 11:1122434. doi: 10.3389/fped.2023.1122434

## Introduction

Recently, Álmos Schranc and colleagues published a most interesting experimental study comparing flow-controlled ventilation (FCV) to pressure-regulated volume-controlled ventilation (PRVC) in a pediatric pig-model of healthy and surfactant depleted, injured lungs (1). This paper provides valuable insights into FCV and associated phenomena at very low tidal volumes. The results show a slightly better and more homogeneous lung aeration in FCV, but inferior gas exchange compared to PRVC. At first sight, this may appear to be strange as better aeration is normally associated with better gas exchange. Notwithstanding this, the authors draw overall positive conclusions regarding the clinical applicability and efficacy of FCV.

Schranc et al. already mentioned differences in dead space which may provide an explanation for the somewhat contradictory results. We fully agree with their assumption and try to give some detailed insights into this issue by replicating the respiratory circuits and calculating the actual dead space effect. In fact, carbon dioxide removal is very susceptible to changes in apparatus dead space, especially in this weight range (2, 3).

## Dead space estimation

**Figure 1:**
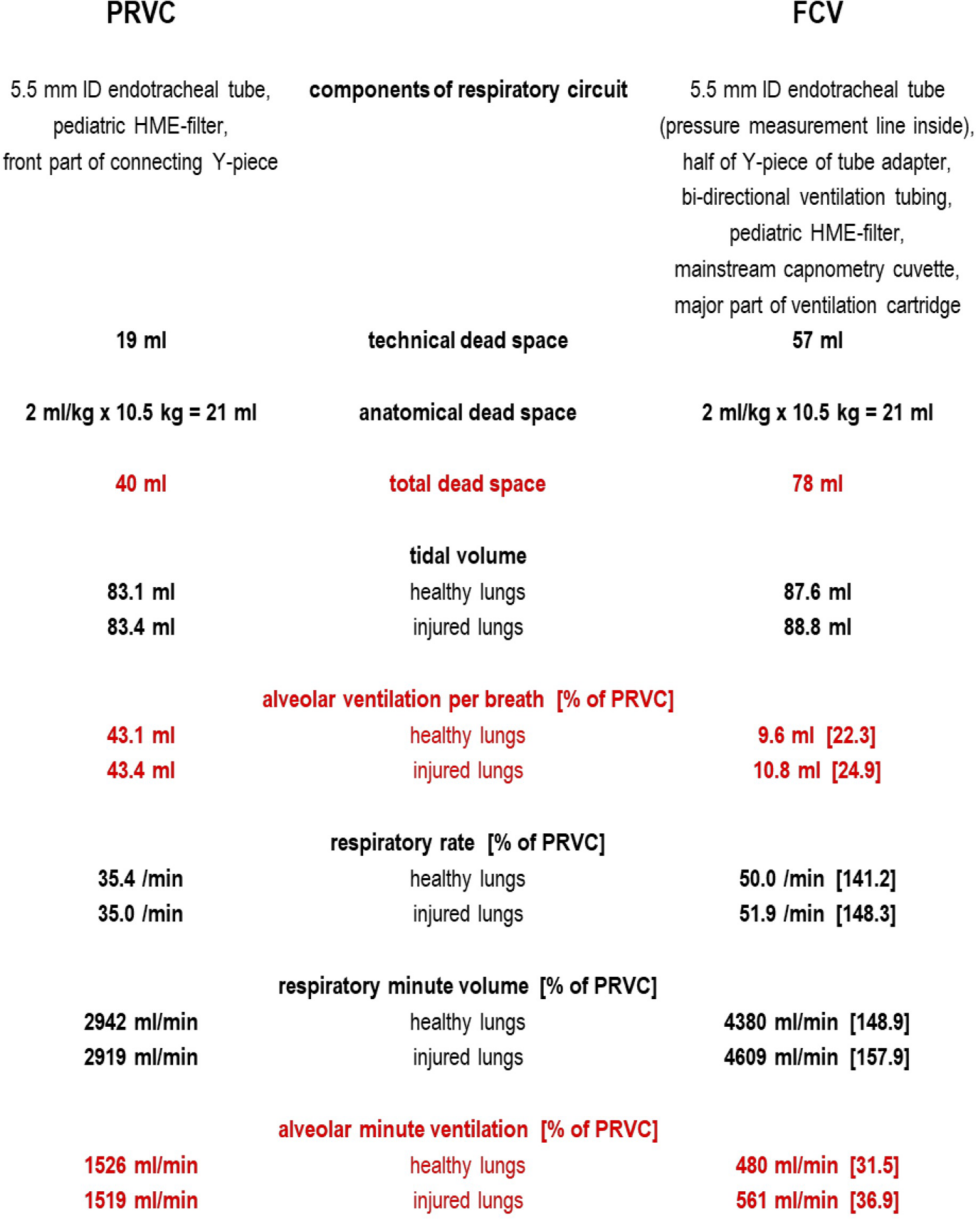
Technical dead space in PRVC and FCV as measured by bubble-free instillation of low surface tension water (mean of three repeated measurements) and calculation of alveolar ventilation based on median values as reported in (1).

In Figure 1 we have listed the parts of the respiratory circuits which geometrically determine the technical dead space. In the methods section Schranc et al. report that the same 5.5 mm inner diameter tube and (as confirmed by them upon inquiry) the same pediatric HME-filter were used in both groups. We consulted the manufacturer's product information on the tube adapter (4) and other components in the FCV system, checked the technical dead space experimentally, and found a technical dead space of 57 ml. In the PRVC system, considering side-stream capnometry was applied via the capnometry port of the pediatric HME-filter as usual in pediatric care, we measured only 19 ml technical dead space.

To estimate alveolar ventilation in both setups one must also consider the anatomical dead space, which is 21 ml (assuming 2 ml/kg as for intubated pediatric patients with a mean piglet weight of 10.5 kg).

The total dead space for FCV was then probably around 78 ml, whereas in PRVC it was only 40 ml. Using mean values for tidal volume and respiratory rate reported by the authors (1), in FCV the alveolar minute ventilation was probably around 480 ml/min (healthy lungs) and 561 ml/min (injured lungs). In contrast, the alveolar minute ventilation in PRVC was likely somewhat larger: 1,526 ml/min (healthy lungs) and 1,519 ml/min (injured lungs).

Two ml/kg anatomical dead space in piglets is probably an underestimate as they have longer bronchi than small children, so alveolar ventilation might have been even less in both groups. This would favor PRVC over FCV even more as cyclic alveolar ventilation would then tend to only a few milliliters in FCV which cannot sufficiently handle the oxygen demand even at an increased FiO_2_ of 0.4 as in the study by Schranc et al.

## Discussion

If our geometrical estimate of the dead space is correct, then the reported performance of FCV is quite remarkable: With about 1/3rd of the alveolar minute ventilation of PRVC, the gas exchange is only slightly worse. Therefore, we fully agree with the overall positive conclusions of the authors on the efficacy of FCV.

Considering the small functional residual capacity of piglets, the substantially lower alveolar ventilation in FCV may very probably have led to a lower alveolar oxygen concentration. In addition, the shorter ventilation cycle time of only 1.2 s in FCV (in contrast to 1.7 s in PRVC) may have also compromised oxygenation. In combination with a higher metabolic rate of piglets leading to a higher oxygen demand, this may provide an explanation for the slightly inferior oxygenation and increased intrapulmonary shunt in FCV, despite better aeration of the lungs.

Because of the lower alveolar minute ventilation in FCV, adequate carbon dioxide removal demanded a remarkably higher respiratory rate and overall minute volume from the ventilator. Unfortunately, this results in elevated levels of applied mechanical power and dissipated energy both of which have become accepted risk parameters for VILI (5).

In contrast to PRVC, FCV is an entirely dynamic ventilation mode without any intracyclic flow pause. Gas flows are fully controlled (effectively constant and preferably identical) over both inspiration and expiration phases (6). This does not only allow for minimization of energy dissipation in the patient (7, 8), it also enables accurate estimation of the dynamic lung compliance curve of the individual patient during ventilation. In turn, this then permits the positive end-expiratory pressure (PEEP) and peak pressure to be titrated (=compliance-guided individualization of ventilator settings), so the patient is ventilated over the whole of the linear portion of the compliance curve, thereby maximizing tidal volume, minimizing effects of dead space, and achieving benefits in terms of aeration and gas exchange (6, 9).

In a study of this nature, it is entirely understandable that the authors opted for comparable tidal volumes in both groups and did not individualize the ventilator settings in FCV to optimize alveolar gas exchange. However, we would like to point out that waiving individualization of FCV ventilation, coupled with the large effect of additional dead space, very probably led to an FCV performance which was substantially suboptimal compared to what it could have been if individualization would have been undertaken.

A possible solution largely reducing the technical dead space would be to place the ejector device currently used to control flows in the FCV ventilator functionally as close as possible to the endotracheal tube (e.g., by a special pediatric ventilation circuit having an inspiratory and expiratory limb with check valves). Thereby, FCV may become applicable in small children and even babies. Currently, according to the manufacturer's instructions, FCV should only be used in patients above 40 kg (10).

## Conclusion

Dead space ventilation differs significantly between groups which must be considered in the interpretation of the results of Schranc et al. (1). Although the use of FCV in pediatrics is currently not intended, the study demonstrates the applicability in principle and, together with our suggestions, may serve as a basis for further investigations.
